# Supplementary material for: Family Physicians Working at Hospitals: A 20-Year Nationwide Trend Analysis in Taiwan
Source: Int J Environ Res Public Health. 2021 Aug 28;18(17):9097. doi: 10.3390/ijerph18179097 (PMC8431456; doi:10.3390/ijerph18179097)
Supplement: Supplementary file 1 [file ijerph-18-09097-s001.zip › ijerph-1332272-supplementary.pdf]

Supplementary materials

Table S1. Changes in number and ratio of physicians registered as family physicians (FPs) in TMA and members enrolled in TAFM in Taiwan from 1999 to 2018.

	Registered FPs in TMA	Changes of FP in TMA/Year	Official members of TAFM	Changes of FP in TAFM/Year	Ratio of FPs in TMA to TAFM
1999	1876		3903		48.1%
2000	1976	100	3947	44	50.1%
2001	2098	122	3996	49	52.5%
2002	2214	116	4062	66	54.5%
2003	2343	129	4102	40	57.1%
2004	2514	171	4159	57	60.4%
2005	2666	152	4228	69	63.1%
2006	2792	126	4282	54	65.2%
2007	2874	82	4371	89	65.8%
2008	2967	93	4459	88	66.5%
2009	3061	94	4565	106	67.1%
2010	3134	73	4676	111	67.0%
2011	3182	48	4786	110	66.5%
2012	3172	-10	4882	96	65.0%
2013	3270	98	4967	85	65.8%
2014	3336	66	5079	112	65.7%
2015	3447	111	5177	98	66.6%
2016	3540	93	5256	79	67.4%
2017	3581	41	5328	72	67.2%
2018	3655	74	5256	-72	69.5%

Table S2. Ratio of family physicians to physicians registered in other specialties in TMA in Taiwan from 1999 to 2018.

	**Family Medicine**	**Internal Medicine**	**Surgery**	**Pediatrics**	**Gynecology**	**Others**	**General Practice**	**Total Physicians**	**Ratio of FPs to other specialties**
**1999**	1876	6497	3079	2256	2165	8371	3715	27,959	6.7%
**2000**	1976	6726	3120	2414	2207	9095	3596	29,134	6.8%
**2001**	2098	6930	3154	2550	2204	9797	3499	30,232	6.9%
**2002**	2214	7024	3198	2620	2184	10,362	3592	31,194	7.1%
**2003**	2343	7148	3208	2666	2179	10,927	3561	32,032	7.3%
**2004**	2514	7300	3225	2725	2172	11,513	3587	33,036	7.6%
**2005**	2666	7504	3219	2760	2140	12,070	3628	33,987	7.8%
**2006**	2792	7768	3258	2780	2149	12,611	3642	35,000	8.0%
**2007**	2874	7980	3340	2817	2168	13,220	3593	35,992	8.0%
**2008**	2967	8289	3397	2895	2184	13,836	3583	37,151	8.0%
**2009**	3061	8392	3433	2978	2180	14,408	3518	37,970	8.1%
**2010**	3134	8545	3489	3004	2179	15,014	3635	39,000	8.0%
**2011**	3182	8676	3565	3062	2174	15,289	4235	40,183	7.9%
**2012**	3172	8658	3463	3090	2189	15,574	5040	41,186	7.7%
**2013**	3270	8796	3489	3171	2216	16,111	5153	42,206	7.7%
**2014**	3336	8933	3537	3273	2237	16,592	5303	43,211	7.7%
**2015**	3447	9090	3582	3358	2262	17,104	5349	44,192	7.8%
**2016**	3540	9283	3631	3455	2322	17,701	5281	45,213	7.8%
**2017**	3581	9409	3679	3537	2373	18,283	5590	46,452	7.7%
**2018**	3655	9721	3830	3619	2412	18,888	5529	47,654	7.7%
